# Evaluation of vascular endothelial growth factor (VEGF) level in the tears and serum of age-related macular degeneration patients

**DOI:** 10.1038/s41598-022-08492-7

**Published:** 2022-03-15

**Authors:** Mohamad Shahidatul-Adha, Embong Zunaina, Mazlan N. Aini-Amalina

**Affiliations:** 1 Department of Ophthalmology and Visual Science, School of Medical Sciences, Universiti Sains Malaysia, 16150 Kubang Kerian, Kelantan Malaysia; 2 Hospital Universiti Sains Malaysia, Jalan Raja Perempuan Zainab II, Kubang Kerian, 16150 Kota Bharu, Kelantan Malaysia

**Keywords:** Biomarkers, Medical research, Pathogenesis, Risk factors

## Abstract

Age-related macular degeneration (AMD) is an important cause of irreversible central blindness worldwide. Clinical manifestations range from asymptomatic in early and intermediate AMD to significant vision loss in late AMD. Approximately 10% of cases of early AMD eventually progress to the late advanced stage, influenced by the upregulation of vascular endothelial growth factor (VEGF). In this study, we evaluated VEGF concentration in the tears and serum of AMD patients. Our study revealed a significantly higher level of VEGF in the tears of patients with AMD compared with controls. The tear VEGF level has high sensitivity and specificity, and is significantly related to the severity of AMD, whilst serum VEGF level is non-specific and non-predictive of AMD severity. Thus, VEGF level in the tears may be used as a non-invasive biomarker for AMD progression. A large cohort study is needed for further verification.

## Introduction

Age-related macular degeneration (AMD) is a major cause of severe visual impairment in aging populations^1^. The prevalence of AMD among Asian and white populations is relatively comparable^2,3^. The projected number of patients with AMD worldwide in 2020 was 196 million, with 1.8 million cases already blind due to late AMD^1^. Late AMD is the advanced stage of the disease in which near task, performance at work, and quality of life are significantly impaired^4,5^. The resultant permanent blindness in late AMD creates a considerable economic burden globally and causes significant psychological, emotional, and financial strains^6,7^.

Dysfunction and degeneration of retinal pigment epithelium (RPE) cells in the aging retina are responsible for AMD development and progression^8–10^. However, the key mediator in its progression to late neovascular or wet AMD is vascular endothelial growth factor (VEGF)^10,11^. VEGF promotes angiogenesis in the initial stage of choroidal neovascularization that further leads to increased vascular permeability^11–13^. Successful anti-VEGF treatment strategies for choroidal neovascularization associated with AMD over the past few decades have proven that VEGF is the molecular basis for wet AMD progression^14,15^.

VEGF level in vitreous and aqueous humor are significantly higher in the AMD group than in the control group^16–18^. However, the quantification of VEGF level in the vitreous and aqueous humor is relatively invasive and inappropriate for a routine study due to potential surgical complication and contamination^19^. Ethically, the procedures are only allowed if performed during cataract or vitreoretinal surgery. A practically easy way to quantify VEGF levels is through blood sampling. A higher mean serum VEGF levels in patients with exudative AMD was observed in previous studies^11,20^. However, serum VEGF levels are also affected and elevated in various systemic diseases and conditions, such as diabetes mellitus (DM), hypertension, hyperlipidaemia, renal disease, and stroke, as well as in smokers^21–25^.

Tear fluid analysis has gained interest in ophthalmology research in recent years^26,27^. It not only reflects the health status of the ocular surface, but it also enables monitoring and understanding of the clinical aspects of many intraocular diseases^13,21,28–30^. The tear film is readily accessible, and collection is easily performed using various methods, such as microcapillary tubes, Schirmer strip, or surgical sponges^31^. There are no prior studies evaluating VEGF levels in the tear fluid of AMD patients. The objective of this study was to evaluate and compare VEGF levels in the tears and serum of patients with AMD.

## Results

### Demographic data

We recruited 108 patients for this study. The study population comprised 72 patients with AMD (early AMD: 36, late AMD: 36) and the control group consisted of 36 patients. The demographic characteristics of the participants and risk factors for AMD are summarized in Table 1.

**Table 1:** Demographic characteristics and systemic profiles of the subjects.

Variables	Total (n = 108)	Early AMD (n = 36)	Late AMD (n = 36)	Control (n = 36)	*p* value
**Age (years)**					
(Mean ± SD)	64.3 ± 8.3	66.6 ± 7.1	68.7 ± 5.1	57.6 ± 8.1	< 0.001^a^
**Sex (n, %)**					
Male	47 (43.5)	15 (41.7)	22 (61.1)	10 (27.8)	0.016^b^
Female	61 (56.5)	21 (58.3)	14 (38.9)	26 (72.2)	
**Race (n, %)**					
Malay	92 (85.2)	29 (80.6)	29 (80.6)	34 (94.4)	0.160^b^
Chinese	16 (14.8)	7 (19.4)	7 (19.4)	2 (5.6)	
**Lifestyle (n, %)**					
Cigarette-smoking					
Active smoker	16 (14.8)	6 (16.7)	6 (16.7)	4 (11.1)	0.038^b^
Ex-smoker	15 (13.9)	3 (8.3)	10 (27.8)	2 (5.6)	
Alcohol use history					
Social drinker	8 (7.4)	1 (2.8)	6 (16.7)	1 (2.8)	0.048^c^
**Comorbidity (n, %)**					
Dyslipidemia	35 (32.4)	14 (38.9)	14 (38.9)	7 (19.4)	0.126^b^
Hypertension	57 (52.8)	19 (52.8)	22 (61.1)	16 (44.4)	0.367^b^
IHD	9 (8.4)	3 (8.3)	3 (8.3)	3 (8.3)	> 0.99^c^
DM	37 (34.3)	13 (36.1)	11 (30.6)	13 (36.1)	0.848^b^
CKD	8 (7.4)	7 (19.4)	1 (2.8)	0 (0)	0.005^c^

AMD: age-related macular degeneration, IHD: ischemic heart disease, DM: diabetes mellitus, CKD: chronic kidney disease.

^a^One-way ANOVA.

^b^Chi-square test.

^c^Fisher’s exact test.

The mean age of the enrolled subjects was 64.3 ± 8.3 years. A significant difference in the mean age was observed between the groups (*p* < 0.001) as follows: lowest in the control group (57.6 ± 8.1 years) and relatively higher in the early AMD (66.6 ± 7.1 years) and late AMD (68.7 ± 5.1 years) groups. Racial distribution showed that 92 patients (85.2%) were Malay and the remaining 14.8% were Chinese. Among the patients with AMD, early AMD was more common in women (58.3%), while late AMD was more frequent in men (61.1%).

Of all the participants, 16 (14.8%) were active smokers during the study period; 12 of them were in the AMD group (6 in early AMD, 6 in late AMD). Eight patients (7.4%) had a history of alcohol consumption, and six of them were in the late AMD group.

Hypertension was the most common comorbidity among participants in each group (61.1% in late AMD, 52.8% in early AMD, and 44.4% in control). In both AMD groups, dyslipidemia (38.9%) was the second most common illness, followed by DM (36.1% in early AMD and 30.6% in late AMD), chronic kidney disease (CKD), and ischemic heart disease (IHD). In the control group, the second most common illness was DM (36.1%), followed by dyslipidemia (19.4%) and IHD (8.3%).

There was a significant association between AMD and smoking (*p* = 0.038), alcohol consumption (*p* = 0.048), and CKD (*p* = 0.005). However, no significant association was found between AMD and dyslipidemia, hypertension, DM, and IHD.

### VEGF levels in serum and tears

The late AMD group showed the highest mean level of tear VEGF (292.88 ± 73.89 pg/mL), followed by the early AMD (161.15 ± 36.73 pg/mL) and control (117.56 ± 16.66 pg/mL) groups. The mean level of VEGF in serum was also the highest in the late AMD group (260.10 ± 76.47 pg/mL) compared with the early AMD (154.90 ± 39.09 pg/mL) and control (152.11 ± 36.08 pg/mL) groups. The mean VEGF levels were significantly different between the three groups in tear (*p* < 0.001) and serum (*p* < 0.001) samples after adjustment for age, sex, race, lifestyle (smoking and alcohol use), and comorbidities (Table 2).

**Table 2:** Comparison of mean VEGF levels between the groups.

VEGF (pg/mL)	Early AMD (n = 36)	Late AMD (n = 36)	Control (n = 36)	F statistics (*df*)	*p* value*
**Tear**					
(Mean ± SD)	161.15 ± 36.73	292.88 ± 73.89	117.56 ± 16.66	84.16 ± 2.98	< 0.001
**Serum**					
(Mean ± SD)	154.90 ± 39.09	260.10 ± 76.47	152.11 ± 36.08	34.12 ± 2.98	< 0.001

*ANCOVA test applied after adjustment for age, sex, race, smoking and alcohol consumption, and comorbidities. *p* < 0.05 was considered significant.

Significant mean differences in tear VEGF between late and early AMD (*p* < 0.001), between late AMD and control (*p* < 0.001), and between early AMD and control (*p* = 0.001) were found after Bonferroni post hoc analysis. There was also a significant mean difference in serum VEGF levels between late and early AMD (*p* < 0.001) and between late AMD and control (*p* < 0.001). There was no significant difference between the early AMD and control groups (*p* > 0.99) (Table 3).

**Table 3:** Post-hoc comparison of mean tear and serum VEGF after adjustment.

Variable	Group	Mean difference (95%, CI)	*p* value*
Tear VEGF	Late AMD versus early AMD	131.72 (103.85, 159.60)	< 0.001
	Late AMD versus control	175.32 (147.45, 203.19)	< 0.001
	Early AMD versus control	43.60 (15.72, 71.46)	0.001
Serum VEGF	Late AMD versus early AMD	105.19 (74.35, 136.03)	< 0.001
	Late AMD versus control	107.98 (77.14, 138.83)	< 0.001
	Early AMD vs control	2.79 (− 28.05, 33.63)	> 0.99

*AMD* Age-related macular degeneration, *VEGF* Vascular endothelial growth factor.

*Bonferroni post-hoc comparison test, *p* < 0.05 was considered significant.

**Figure 1:**
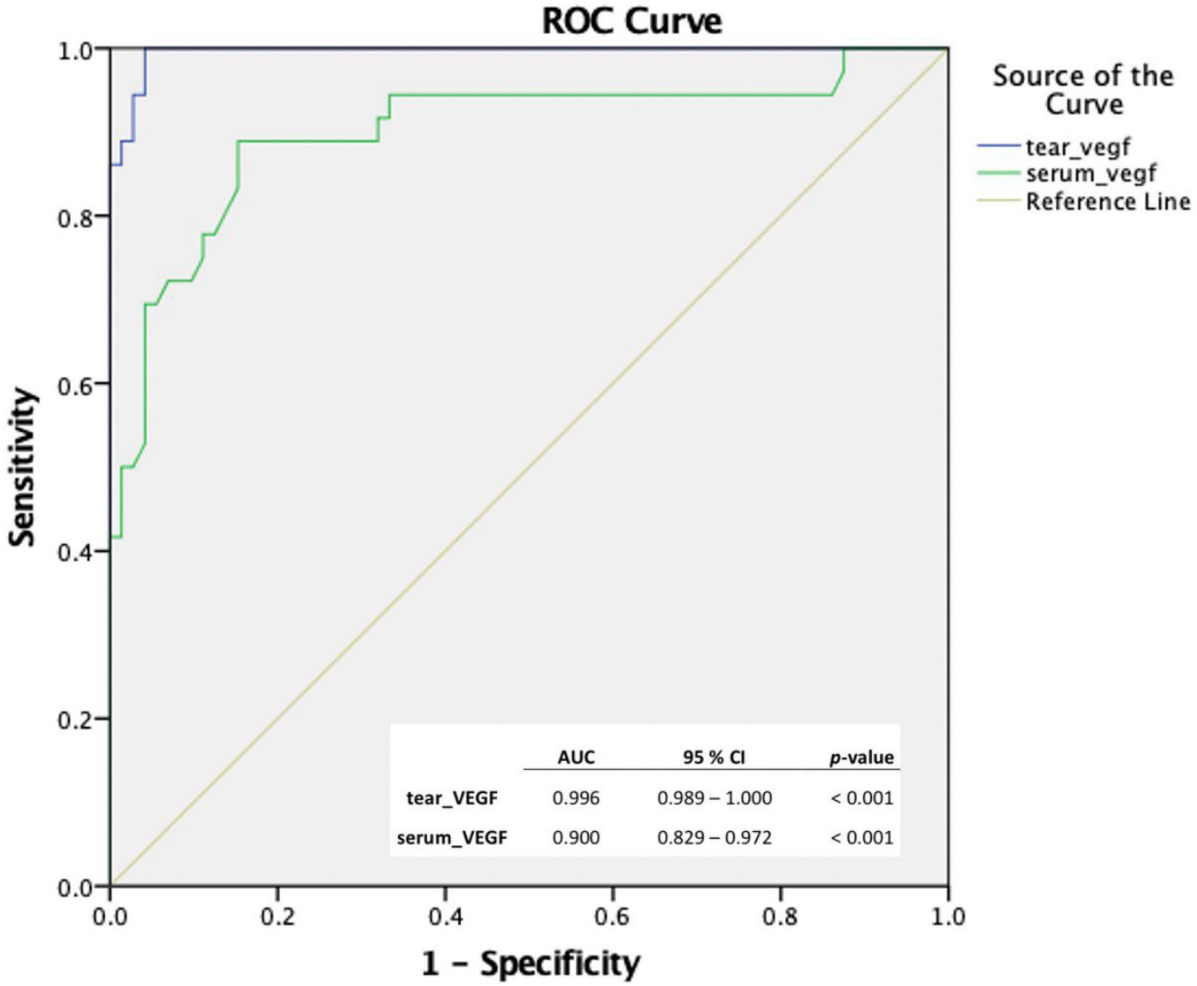
ROC curves represent predictive value of VEGF level in determining AMD severity. The tear VEGF curve shows an excellent AUC (0.996), indicating highly sensitive and specific value of the tear VEGF as a predictor for AMD severity. There are diagonal segments in the serum VEGF curve produced by ties between the positive and the negative actual state group; and statistics may be biased despite the excellent AUC (0.900) observed. *ROC* Receiver operating characteristic, *VEGF* Vascular endothelial growth factor, *AMD* Age-related macular degeneration, *AUC* Area under ROC curve.

The clinical sensitivity and specificity of tear and serum VEGF level in predicting AMD severity is shown in Fig. 1. The observed area under the receiver operating characteristic (ROC) curve (AUC) was excellent for tear VEGF (AUC = 0.996). An excellent AUC was also observed for serum VEGF, however there are diagonal segments in that curve produced by ties between the positive and the negative actual state group; statistics may be biased.

### Associated factors for VEGF levels in serum and tears

The associated factors for tears and serum VEGF levels among AMD patients are summarized in Table 4. Based on simple linear regression analysis for tear VEGF, age, sex, race, alcohol use, CKD, and serum VEGF level were the significant variables (p < 0.25) among the associated factors. These six significant variables were included in the multiple linear regression analysis. Smoking status, hypertension, and dyslipidemia were also included as they are clinically important. The results showed age (*p* = 0.003) and serum VEGF level (*p* < 0.001) as significant factors associated with tear VEGF.

**Table 4:** Factors associated with tear and serum VEGF among AMD patients.

Variables	Regression coefficient (*b*)	*t*-stats	*p* value	Regression coefficient (*b*)	*t*-stats	*p* value
Tear VEGF	Simple linear regression*			Multiple linear regression**		
Age	4.624	4.939	< 0.001	2.125	3.086	0.003
Sex	− 28.315	− 1.652	0.101	0.006	0.001	> 0.99
Race	37.796	1.578	0.117	− 12.047	− 0.596	0.553
Smoking status	16.974	1.062	0.291	− 10.759	− 1.084	0.281
Alcohol use	− 82.940	− 2.603	0.011	− 47.572	− 1.710	0.090
Hypertension	− 19.518	− 1.139	0.257	15.775	1.334	0.183
Dyslipidemia	− 6.911	− 0.376	0.708	− 9.205	− 0.757	0.451
DM	9.890	0.546	0.586			
IHD	− 3.544	− 0.114	0.990			
CKD	50.589	1.557	0.122	22.250	1.134	0.260
Serum VEGF	0.958	13.246	< 0.001	0.875	11.263	< 0.001
**Serum VEGF**						
Age	3.145	3.931	< 0.001	− 0.200	− 0.342	0.783
Sex	− 23.328	− 1.651	0.102	− 5.515	− 0.613	0.541
Race	17.492	0.879	0.381			
Smoking status	26.025	2.001	0.048	16.517	1.998	0.048
Alcohol use	− 45.793	− 1.713	0.090	5.454	0.310	0.757
Hypertension	− 29.271	− 2.102	0.038	− 10.107	− 0.972	0.333
Dyslipidemia	− 7.300	− 0.482	0.631	10.719	1.051	0.296
DM	− 15.73	− 1.058	0.293	− 20.515	− 2.016	0.046
IHD	5.677	0.221	0.825			
CKD	27.445	1.018	0.311			
Tear VEGF	0.651	13.246	< 0.001	0.650	11.810	< 0.001

*DM* Diabetes mellitus, *IHD* Ischemic heart disease, *CKD* Chronic kidney disease, *VEGF* Vascular endothelial growth factor.

*Simple linear regression; *p* value < 0.25 was considered significant.

**Multiple linear regression; *p* value < 0.05 was considered significant. Enter, Stepwise, Backward, and Forward analyses were applied.

For serum VEGF level, age, sex, smoking, alcohol, hypertension, and tear VEGF were the significant variables (*p* < 0.25) based on simple linear regression. Hypertension, dyslipidemia, and DM were also included in the multiple linear regression analysis because of their clinical importance. The significant factors associated with serum VEGF levels were smoking (*p* = 0.048), DM (*p* = 0.046), and tear VEGF level (*p* < 0.001).

### Correlation of VEGF level in tears and serum

**Figure 2:**
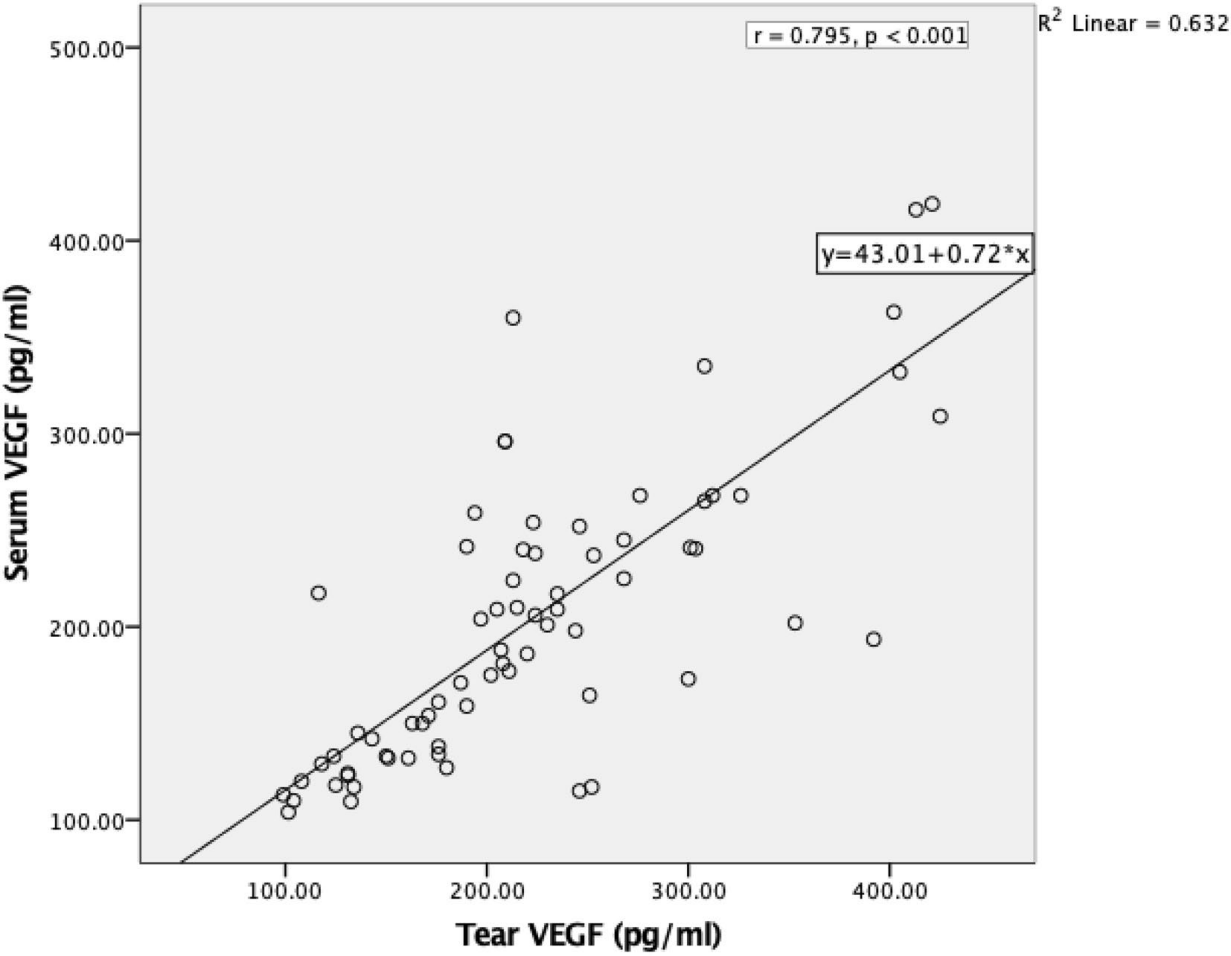
Pearson correlation analysis shows that the tear VEGF level in AMD patients was positively correlated to the serum level (*r* = 0.795, *p* < 0.001).

The relationship between tear and serum VEGF levels among patients with AMD was analyzed using Pearson’s correlation. There was a significant linear and strong correlation between tear and serum VEGF levels (*r* = 0.795, *p* < 0.001) among AMD patients as shown in Fig. 2.

## Discussion

In this study, we evaluated VEGF levels in the tears and serum of AMD patients. Schirmer strip was used for tear fluid collection. The Schirmer strip collection method is a simple procedure, cheap, well tolerated, and relatively painless, with minimal to no discomfort; hence, it is considered superior to the other tear fluid collection methods, including microcapillary tubes, surgical sponges, cellulose acetate filters, rods, spatulas, glass rods, and microsponges^31–35^. The major drawback of Schirmer strips is that the collected volume of tears is not readily available, and there is a need for post-processing procedure, which may alter the final protein concentration^33,35^.

We extracted the tears from the Schirmer strip by a simple dilution over 30 min with assay buffer A, according to the VEGF ELISA kit protocol (LEGEND MAX™, BioLegend, Inc). A preliminary test was performed to validate the extraction method and to ensure reliability and repeatability of the technique. We found a significantly higher level of VEGF in the late exudative AMD compared with the early group, which suggests an active role of VEGF in AMD progression and neovascularization. To the best of our knowledge, our study is the first to evaluate tear VEGF levels in patients with AMD. Therefore, there are no data to which our results can be compared.

Unlike tear fluid, the VEGF content in aqueous and vitreous humor has been extensively studied. A previous study reported a significantly higher mean vitreous VEGF level among patients with AMD than among controls^11^. Diminished blood flow to the retina among AMD patients due to the deposition of drusen in basal laminar and basal linear layer impairs the oxygen and glucose transport through the choriocapillaris^38^. The resultant hypoxic retina triggers an excessive production of VEGF in the eyes of AMD patients, leading to elevated VEGF levels in the vitreous and aqueous humor^12,13^. Our study lacks vitreous and aqueous samples for comparison. However, a recent in-depth analysis of tear fluid using the Schirmer collection method revealed eight similar proteins previously found in the aqueous and vitreous humor, recognized exclusively for AMD^36^. We believe that the tear VEGF level reflects the ocular level of VEGF due to closer proximity and similar circulation^37^.

Predictably, we observed a significantly high mean serum VEGF level in the late AMD after adjusting for age, sex, race, smoking, alcohol consumption, and comorbidities. This is in line with a previous study that reported higher mean serum VEGF levels in AMD patients than in controls^11^. This result indicates a significant association between AMD and serum VEGF levels but is less specific as circulating VEGF levels may be affected by various medical illnesses common in the elderly. Multicenter studies have confirmed that predisposing hypoxic conditions, such as asthma, hypertension, heart failure, atherosclerosis, renal failure, and stroke, as well as smoking and alcoholic consumption, may result in the elevation of serum VEGF levels^21–25,39–42^.

Further analysis showed a significant positive correlation between tear and serum VEGF levels. In addition, we also successfully determined a cut-off value of the tear VEGF to predict the severity of AMD; early AMD, 128 pg/mL (sensitivity 1.000, specificity 0.528), and late AMD, 208.5 pg/mL (sensitivity 1.000, specificity 0.958). We were unable to determine the cut-off value of the serum VEGF in view of probable statistical bias as shown in Fig. 1.

Apart from evaluating and comparing tear and serum VEGF levels, our cross-sectional analysis found an equal number of males and females among the AMD patients, with a higher male proportion in the late AMD group. This is consistent with the previously reported higher prevalence of late AMD among men in the Asian population^2^. In the study, cigarette smoking, alcohol consumption, and CKD were the significant risk factors for AMD. The higher prevalence of smoking among Asian men could explain the impact of smoking on the Asian AMD population^2^. The significance of alcohol and CKD in our AMD patients is supported by previous studies, which reported that a higher risk of developing early AMD could be related to even moderate alcohol consumption and underlying CKD^43,44^.

In the extension of our study, we found that age was a significant factor for tear VEGF, whereas, for serum VEGF, the significant factors were smoking and DM. We found no association between AMD and hypertension or dyslipidemia, although these two risk factors were consistently identified in AMD studies from neighboring countries^2,3,45,46^.

There are several limitations in our study, whereby our results cannot be generalized to other world populations due to a single-center design, and unequal racial distribution. In addition, residual confounding is still possible despite our strict selection criteria, given that AMD is a disease of the elderly, who often have comorbidities. It is difficult to eliminate all confounders for circulating VEGF as the number of eligible subjects for this study will become much smaller. Furthermore, we were unable to detect any difference in the VEGF levels of the two different forms of late AMD due to the limited number of geographic atrophy (GA) in our study population; therefore, GA was not studied. We also limited our study to VEGF detection due to a limited budget.

In the future, multicenter and multiracial studies should be performed for a larger study population. We recommend a concurrent evaluation of VEGF levels in the tears, aqueous and vitreous humor, to enhance our understanding of the relationship between intraocular and ocular surface VEGF levels. Detection of other biomarkers, such as CD146 and tumor necrosis factor-α, as well as other proteomes, may also be considered for a better understanding of AMD pathogenesis and its progression^47,48^.

In summary, our study revealed a significantly higher level of VEGF in the tears of patients with AMD, which was the highest in the late exudative group. The tear VEGF level has high sensitivity and specificity, and is significantly related to the severity of AMD, whilst serum VEGF level is non-specific and non-predictive of AMD severity. Thus, VEGF level in the tears may be used as a non-invasive biomarker for AMD progression. A large cohort study is needed for further verification.

## Methods

### Participation and selection criteria

A comparative cross-sectional study was conducted in Hospital Universiti Sains Malaysia, a tertiary hospital in northeast Malaysia, from October 2016 to September 2018. This study was approved by the local Human Research Ethics Committee of Universiti Sains Malaysia, [Jawatankuasa Etika Penyelidikan Manusia (JEPeM); Registration number: 16080264] and followed the tenets of the Declaration of Helsinki. Informed and written consent was obtained from all patients. This article does not contain any personal information that could lead to the identification of the patients.

The sample size was calculated using G Power 3.1.9. Patients aged ≥ 45 years with AMD were recruited using the non-probability sampling method. The control group comprised individuals aged ≥ 45 years, without any features of AMD. Based on the Wisconsin Age-Related Maculopathy Grading System (WARMGS), early AMD was defined as any retinal drusen or pigmentary changes, whereas late AMD was defined as the presence of GA or neovascular features^14^. The graded characteristics of early AMD are the presence of soft indistinct or hard drusen with pigmentary abnormalities, while the presence of either GA or evidence of exudative features, such as serous sensory retinal detachment, subretinal hemorrhage, or subretinal fibrosis, indicate late AMD.

For both AMD and control groups, patients who had previous laser photocoagulation, photodynamic therapy, intravitreal anti-VEGF injection, or oral supplementation for AMD were excluded. Other exclusion criteria were: (a) refractive error >  − 5.00 DS; (b) pathological myopia with myopic maculopathy; (c) vitreoretinal pathology and macular dystrophies; (d) recent intraocular surgery (within 6 months) prior to the recruitment period; (e) history of any ocular surface or corneal pathology; and (f) any prior history of intraocular inflammatory and infectious diseases such as uveitis and chorioretinitis. Patients with a history of central serous chorioretinopathy and cystoid macular edema of any cause were also excluded. Patients with uncontrolled hypertension, diabetes, and other medical problems with possible high circulating serum VEGF levels related to systemic hypoxia and chronic inflammation were additionally excluded.

We screened a total of 168 patients: 128 AMD and 40 control. Among patients with AMD, 70 had early AMD features, 44 had late features, while another 14 had early feature in one eye and late feature in the opposite eye. However, only 108 patients (36 early AMD, 36 late AMD, and 36 control) who fulfilled the selection criteria were included. There were five patients with GA, although three did not fulfill the inclusion criteria. Considering the limited cases of GA, only the exudative type of late AMD was included in this study.

### Data collection

Demographic data (age, race, and sex), smoking status (active, ex-smoker, and non-smoker), history of alcohol consumption, and systemic illnesses (hypertension, dyslipidemia, DM, IHD, and CKD) were obtained from patients through direct questioning and verified from their medical records. A comprehensive ophthalmic assessment was performed by the primary investigator, which included best-corrected visual acuity, slit lamp, and dilated fundus examination. All patients were required to have their fundus photographed, and based on WARMGS, they were classified into early and late AMD.

Patients who had normal fundus photographs without signs of early or late AMD were included in the control group. Both eyes were thoroughly examined. For AMD patients, only one eye with the worst severity was selected for tear sampling. For the control group, the right eye was selected for standardization. All blood and tear sample collections were performed by one designated staff member who was not involved in the study.

### Tear and serum collection

After a detailed explanation of the procedures, tears were collected, followed by blood collection. Patients sat upright in a comfortable position with a well-rested back and head. Basal tears were collected using a diagnostic ophthalmic filter paper (Schirmer strips). One to two drops of topical anesthesia (Alcain® 0.5%, Alcon-, Belgium) were instilled into the selected eye 5 min prior to tear sample collection. The Schirmer strip was then inserted and placed at the outer third of the lower eyelid until the tear wet the strip at least 10 mm over 5 min. The paper strip was then removed from the eye and kept inside a clear 0.5 mL polypropylene bullet tube.

For blood sampling, a 23 G needle attached to a 5 mL syringe was inserted through the skin into a superficial vein in the cubital fossa of the forearm. Blood (3.5 mL) was withdrawn and collected in a serum separator tube. The blood samples were centrifuged at 2500 rpm for 5 min. Serum from the topmost layer was transferred to a bullet container.

All tear and serum samples were immediately sent to the Physiology Laboratory in Hospital USM and stored at − 80 °C during the recruitment process. The serum sample was thawed for 2 h prior to analysis. The tear sample was extracted from the Schirmer strip by dilution with 100 μL Assay buffer A, followed by VEGF quantification without further handling.

### VEGF quantification

A human VEGF ELISA kit with a pre-coated plate (LEGEND MAX™, BioLegend, Inc.) was used to measure the free-form VEGF levels in tears and serum samples. All samples were processed according to the kit protocol in the Biomedical Laboratory and Central Research Laboratory of USM. The initial step involved the addition of 50 μL of Assay Diluent D into 50 μL of sample (serum and tear) to the appropriate wells, which were sealed and incubated for 2 h. The subsequent steps involved the addition of human VEGF detection antibody solution, followed by avidin-HRP solution and substrate solution F. The wells containing human VEGF turned blue. The reaction was stopped by adding stop solution, indicated by color changes to yellow. Duplicate tests were performed for every sample.

Throughout the procedures, the incubation was performed at room temperature, followed by washing four to five times with wash buffer solutions in between steps. The plates were analyzed using a microplate reader at a wavelength of 450–570 nm.

### Statistical analysis

Statistical analyses were performed using the Statistical Package for Social Sciences (SPSS) software, version 24 (IBM, Armonk, NY). All values were tested for normal distribution and equal variances. The data were normally distributed based on the Kolmogorov–Smirnov test. Descriptive data for the AMD and control groups were analyzed using one-way ANOVA, chi-square test, and Fisher’s exact test and are represented as mean values and standard deviations or as numbers and percentages as appropriate.

To compare tear and serum VEGF levels between the early AMD, late AMD, and control groups, the ANCOVA test was used adjusted for age, sex, smoking status, alcohol use, and systemic illness (hypertension, dyslipidemia, DM, IHD, and CKD). The Bonferroni post-hoc test was used to compare tear and serum VEGF levels between the three groups of patients. The factors influencing tear and serum VEGF levels were determined using simple and multiple linear regression analyses. A *p* value of < 0.05 was considered significant. A receiver operating characteristic (ROC) curve was generated to determine the predictive value of the obtained tear and serum VEGF level based on their sensitivity and specificity in patients with AMD. Pearson’s correlation was used to evaluate correlations between serum and tear VEGF; *r* was graded as follows: *r* < 0.25, little or no correlation; *r* = 0.25–0.50 fair correlation; *r* = 0.50–0.75 moderate/good correlation; and *r* > 0.75 excellent correlation.

## Data Availability

All relevant data are presented in this paper. Additional information and study material are available from the corresponding author upon request.
